# Therapeutic hypothermia for neonatal encephalopathy: a report from the first 3 years of the Baby Cooling Registry of Japan

**DOI:** 10.1038/srep39508

**Published:** 2017-01-04

**Authors:** Kennosuke Tsuda, Takeo Mukai, Sachiko Iwata, Jun Shibasaki, Takuya Tokuhisa, Tomoaki Ioroi, Hiroyuki Sano, Nanae Yutaka, Akihito Takahashi, Akihito Takeuchi, Toshiki Takenouchi, Yuko Araki, Hisanori Sobajima, Masanori Tamura, Shigeharu Hosono, Makoto Nabetani, Osuke Iwata, The Baby Cooling Registry of Japan Collaboration Team, Hiroyuki Adachi, Satoru Aiba, Shinnosuke Akiyoshi, Takasuke Amizuka, Mikihiro Aoki, Hirokazu Arai, Junichi Arai, Hideomi Asanuma, Atsushi Baba, Motoki Bonno, Yusuke Daimon, Tomoko Egashira, Rie Fukuhara, Naoki Fukushima, Masahide Futamura, Sayaka Harada, Tsukasa Hattori, Nobuhide Henmi, Takehiko Hiroma, Tadashi Hisano, Kuniko Ieda, Koichi Iida, Shigeo Iijima, Ken Imai, Takashi Imamura, Shinkai Inoue, Akio Ishiguro, Keiji Suzuki, Tsutomu Ishii, Takashi Ito, Masanori Iwai, Shinnichiro Iwataki, Wataru Jinnai, Akihiko Kai, Taro Kanbe, Masahiro Kinoshita, Hiroshi Kanda, Masatoshi Kaneko, Akihiko Kawase, Hitoshi Kawato, Yoshikazu Kida, Minako Kihara, Hiroyuki Kitano, Makoto Kishigami, Naoaki Shibata, Osamu Kito, Akira Kobayashi, Yoshinori Kohno, Minoru Kokubo, Masatoshi Kondo, Eri Konishi, Masaki Kugo, Masanori Kouwaki, Takeshi Kumagai, Takashi Kusaka, Takeshi Kusuda, Tomoki Maeda, Yoshinobu Maede, Tomoaki Maji, Tomoko Makiya, Kennichi Maruyama, Ken Masunaga, Atsushi Matsumoto, Hiroshi Matsumoto, Naoko Matsumoto, Aya Mima, Kyoko Minagawa, Yoshihiro Minosaki, Hideki Minowa, Mazumi Miura, Masafumi Miyata, Yayoi Miyazono, Hiroshi Mizumoto, Kazuhiro Mori, Ichiro Morioka, Takeshi Morisawa, Ken Nagaya, Yoshihisa Nagayama, Atsushi Naito, Kenji Nakamura, Makoto Nakamura, Atsushi Nakao, Hideto Nakao, Yusuke Nakazawa, Yutaka Nishimura, Naoto Nishizaki, Kazuhiko Nosaka, Masatoshi Nozaki, Masayuki Ochiai, Atsushi Ohashi, Shigeru Ohki, Isaku Omori, Yoshiteru Osone, Junko Saito, Yoshiaki Sato, Yoshitake Sato, Kazuo Seki, Yoshitsugu Shirakawa, Hiroyuki Shiro, Hiroshi Suzumura, Ritsuko Takahashi, Yasushi Takahata, Atsuko Taki, Taihei Tanaka, Itaru Tateishi, Mikio Tsunei, Touhei Usuda, Yukari Yada, Junko Yamamoto, Masahito Yamamoto, Hitoshi Yoda, Akiko Yokoi, Shinobu Yoshida, Taketoshi Yoshida, Tomohide Yoshida, Kayo Yoshikawa

**Affiliations:** 1 Department of Paediatrics and Child Health, Kurume University School of Medicine, Fukuoka, Japan; 2 Center for Advanced Medical Research, Institute of Medical Science, University of Tokyo, Tokyo, Japan; 3 Centre for Developmental and Cognitive Neuroscience, Kurume University School of Medicine, Fukuoka, Japan; 4 Department of Neonatology, Kanagawa Children’s Medical Center, Kanagawa, Japan; 5 Division of Neonatology, Perinatal Medical Center, Kagoshima City Hospital, Kagoshima, Japan; 6 Department of Pediatrics, Perinatal Medical Center, Himeji Red Cross Hospital, Hyogo, Japan; 7 Department of Pediatrics, Yodogawa Christian Hospital, Osaka, Japan; 8 Department of Pediatrics, Kurashiki Central Hospital, Okayama, Japan; 9 Division of Neonatology, National Hospital Organization Okayama Medical Center, Okayama, Japan; 10 Department of Pediatrics, Keio University School of Medicine, Tokyo, Japan; 11 Faculty of Informatics, Shizuoka University, Shizuoka, Japan; 12 Division of Neonatology, Center for Maternal, Fetal and Neonatal Medicine, Saitama Medical Center, Saitama Medical University, Saitama, Japan; 13 Division of Neonatology, Nihon University Itabashi Hospital, Tokyo, Japan; 14 Department of Pediatrics, Akita University Hospital, Akita, Japan; 15 Department of Pediatrics, Yamagata Prefectural Central Hospital, Yamagata, Japan; 16 Division of Neonatology, Ehime Prefectural Central Hospital, Ehime, Japan; 17 Division of Neonatology, Aomori Prefectural Central Hospital, Aomori, Japan; 18 Department of Pediatrics, National Hospital Nagasaki Medical Center, Nagasaki, Japan; 19 Division of Neonatology, Japanese Red Cross Akita Hospital, Akita, Japan; 20 Division of Neonatology, Ibaraki Children’s Hospital, Ibaraki, Japan; 21 Division of Neonatology, Hokkaido Medical Center for Child Health and Rehabilitation, Hokkaido, Japan; 22 Department of Pediatrics, Shinshu University Hospital, Nagano, Japan; 23 Division of Neonatology, National Hospital Organization Miechuo Medical Center, Mie, Japan; 24 Department of Pediatrics, Tomakomai City Hospital, Hokkaido, Japan; 25 Department of Pediatrics, National Hospital Organization Saga Hospital, Saga, Japan; 26 Division of Neonatology, Hiroshima Prefectural Hospital, Hiroshima, Japan; 27 Division of Neonatology, Almeida Memorial Hospital, Oita, Japan; 28 Department of Pediatrics, Aichi Medical University Hospital, Aichi, Japan; 29 Department of Neonatology, Osaka City General Hospital, Osaka, Japan; 30 Division of Neonatology, Sapporo City General Hospital, Hokkaido, Japan; 31 Division of Neonatal Intensive Care Unit, Tokyo Women’s Medical University Medical Center East, Tokyo, Japan; 32 Division of Neonatology, Nagano Children’s Hospital, Nagano, Japan; 33 Division of Neonatology, St.Mary’s Hospital, Fukuoka, Japan; 34 Division of Neonatology, Tosei General Hospital, Aichi, Japan; 35 Division of Neonatology, Oita Prefectural Hospital, Oita, Japan; 36 Perinatal Center, Hamamatsu University School of Medicine, Shizuoka, Japan; 37 Department of Neonatology, Tokyo Women’s Medical University, Tokyo, Japan; 38 Department of Pediatrics, Takeda General Hospital, Oita, Japan; 39 Department of Pediatrics, Fukuoka University Hospital, Fukuoka, Japan; 40 Department of Pediatrics, Tokai University Hospital, Kanagawa, Japan; 41 Department of Pediatrics, Fukushima National Hospital, Fukushima, Japan; 42 Department of Pediatrics, Kitasato University Hospital, Kanagawa, Japan; 43 Department of Pediatrics, Kumamoto University Hospital, Kumamoto, Japan; 44 Department of Pediatrics, Onomichi Hospital, Hiroshima, Japan; 45 Division of Neonatology, Shikoku Medical Center for Children and Adults, Kagawa, Japan; 46 Department of Pediatrics, Aizenbashi Hospital, Osaka, Japan; 47 Department of Pediatrics, Sasebo City General Hospital, Nagasaki, Japan; 48 Division of Neonatology, Iizuka Hospital, Fukuoka, Japan; 49 Department of Pediatrics, Fukushima Medical University, Fukushima, Japan; 50 Division of Neonatology, Kumamoto City Hospital, Kumamoto, Japan; 51 Division of Neonatology, Narita Red Cross Hospital, Chiba, Japan; 52 Division of Neonatology, Matsudo City Hospital, Chiba, Japan; 53 Division of Neonatology, Japanese Red Cross Kyoto Daiichi Hospital, Kyoto, Japan; 54 Division of Neonatology, Ishikawa Prefectural Central Hospital, Ishikawa, Japan; 55 Department of Pediatrics, Shimane University School of Medicine, Shimane, Japan; 56 Division of Neonatology, Japanese Red Cross Nagoya First Hospital, Aichi, Japan; 57 Department of Pediatrics, Nagaoka Red Cross Hospital, Niigata, Japan; 58 Division of Neonatology, Gifu Prefectural General Medical Center, Gifu, Japan; 59 Department of Pediatrics, Kainan Hospital, Aichi, Japan; 60 Division of Neonatology, Tokyo Metropolitan Children’s Medical Center, Tokyo, Japan; 61 Department of Pediatrics, Matsue Red Cross Hospital, Shimane, Japan; 62 Division of Neonatology, Toyohashi Municipal Hospital, Aichi, Japan; 63 Neonatal Intensive Care Unit, Department of Pediatrics, Wakayama Medical University Hospital, Wakayama, Japan; 64 Division of Neonatology, Kagawa University Hospital, Kagawa, Japan; 65 Department of Pediatrics, Yamaguchi University Hospital, Yamaguchi, Japan; 66 Department of Pediatrics, Oita University Hospital, Oita, Japan; 67 Department of Pediatrics and Neonatology, Japan Red Cross Ise Hospital, Mie, Japan; 68 Division of Neonatology, Okinawa Prefectural Chubu Hospital, Okinawa, Japan; 69 Division of Neonatology, Gunma Children’s Medical Center, Gunma, Japan; 70 Division of Neonatology, Tokyo Metropolitan Ohtsuka hospital, Tokyo, Japan; 71 Department of Pediatrics, Iwate Medical University, Iwate, Japan; 72 Division of Neonatology, Asahi General Hospital, Chiba, Japan; 73 Department of Pediatrics, Kitakyushu Municipal Medical Center, Fukuoka, Japan; 74 Department of Pediatrics, Hyogo College of Medicine College Hospital, Hyogo, Japan; 75 Neonatal Center, Kawaguchi Municipal Medical Center, Saitama, Japan; 76 Division of Neonatology, Nara Prefecture General Medical Center, Nara, Japan; 77 Division of Pediatrics and Perinatology, Tottori University Faculty of Medicine, Tottori, Japan; 78 Department of Pediatrics, Fujita Health University School of Medicine, Aichi, Japan; 79 Department of Child Health, Faculty of Medicine University of Tsukuba, Ibaraki, Japan; 80 Department of Pediatrics, Kitano Hospital, Hyogo, Japan; 81 Department of Pediatrics, Tokushima Prefectural Central Hospital, Tokushima, Japan; 82 Department of Pediatrics, Kobe University Hospital, Hyogo, Japan; 83 Department of Pediatrics, Kakogawa West City Hospital, Hyogo, Japan; 84 Division of Neonatal Intensive Care Unit, Asahikawa Medical University Hospital, Hokkaido, Japan; 85 Division of Neonatology, Niigata City General Hospital, Niigata, Japan; 86 Division of Neonatology, Yamanashi Prefectural Central Hospital, Yamanashi, Japan; 87 Division of Neonatology, Japanese Red Cross Otsu Hospital, Shiga, Japan; 88 Division of Neonatology, Japanese Red Cross Medical Center, Tokyo, Japan; 89 Division of Neonatology, Hyogo Prefectural Kobe Children’s Hospital, Hyogo, Japan; 90 Division of Neonatology, Hiroshima City Hiroshima Citizens Hospital, Hiroshima, Japan; 91 Perinatal Medical Center (NICU), Juntendo University Urayasu Hospital, Tokyo, Japan; 92 Division of Neonatology, Fukui Prefectural Hospital, Fukui, Japan; 93 Department of Neonatal Medicine, Osaka Medical Center and Research Institute for Maternal and Child Health, Osaka, Japan; 94 Department of Pediatrics, Kyushu University Hospital, Fukuoka, Japan; 95 Department of Pediatrics, Kansai Medical University Hospital, Osaka, Japan; 96 Division of Neonatology, Seirei Hamamatsu General Hospital, Shizuoka, Japan; 97 Division of Neonatology, Tokyo Metropolitan Bokutoh Hospital, Tokyo, Japan; 98 Division of Neonatology, Kimitsu Chuo Hospital, Chiba, Japan; 99 Division of Neonatology, Miyagi Children’s Hospital, Miyagi, Japan; 100 Division of Neonatology, Center for Maternal-Neonatal Care, Nagoya University Hospital, Aichi, Japan; 101 Department of Pediatrics, Ota Memorial Hospital, Toyama, Japan; 102 Division of Neonatology, Yokohama City University Medical Center, Kanagawa, Japan; 103 Division of Neonatology, Fukuoka Shin Mizumaki Hospital, Fukuoka, Japan; 104 Division of Neonatology, Yokohama Rosai Hospital, Kanagawa, Japan; 105 Department of Pediatrics, Dokkyo Medical University Hospital, Tochigi, Japan; 106 Division of Neonatology, Japanese Red Cross Sendai Hospital, Miyagi, Japan; 107 Division of Neonatology, Fukuoka Children’s Hospital, Fukuoka, Japan; 108 Division of Neonatology, Tokyo Medical and Dental University, University Hospital of Medicine, Tokyo, Japan; 109 Division of Neonatology, Japanese Red Cross Nagoya Daini Hospital, Aichi, Japan; 110 Department of Pediatrics, Saiseikai Yokohamashi Tobu Hospital, Kanagawa, Japan; 111 Department of Pediatrics, Tottori Prefectural Central Hospital, Tottori, Japan; 112 Maternal and Perinatal Center, Niigata University Medical & Dental Hospital, Niigata, Japan; 113 Division of Neonatology, Jichi Medical University Hospital, Tochigi, Japan; 114 Division of Neonatology, Japan Community Healthcare Organization Kyushu Hospital, Fukuoka, Japan; 115 Department of Pediatrics, Nagahama Red Cross Hospital, Shiga, Japan; 116 Division of Neonatology, Toho University Omori Medical Center, Tokyo, Japan; 117 Department of Pediatrics, Nagoya City West Medical Center, Aichi, Japan; 118 Department of Pediatrics, Omihachiman Community Medical Center, Shiga, Japan; 119 Department of Pediatrics, Toyama University Hospital, Toyama, Japan; 120 Department of Pediatrics, University of the Ryukyus Hospital, Okinawa, Japan

## Abstract

Therapeutic hypothermia is recommended for moderate and severe neonatal encephalopathy, but is being applied to a wider range of neonates than originally envisaged. To examine the clinical use of therapeutic hypothermia, data collected during the first 3 years (2012–2014) of the Baby Cooling Registry of Japan were analysed. Of 485 cooled neonates, 96.5% were ≥36 weeks gestation and 99.4% weighed ≥1,800 g. Severe acidosis (pH < 7 or base deficit ≥16 mmol/L) was present in 68.9%, and 96.7% required resuscitation for >10 min. Stage II/III encephalopathy was evident in 88.3%; hypotonia, seizures and abnormal amplitude-integrated electroencephalogram were observed in the majority of the remainder. In-hospital mortality was 2.7%; 90.7% were discharged home. Apgar scores and severity of acidosis/encephalopathy did not change over time. The time to reach the target temperature was shorter in 2014 than in 2012. The proportion undergoing whole-body cooling rose from 45.4% to 81.6%, while selective head cooling fell over time. Mortality, duration of mechanical ventilation and requirement for tube feeding at discharge remained unchanged. Adherence to standard cooling protocols was high throughout, with a consistent trend towards cooling being achieved more promptly. The mortality rate of cooled neonates was considerably lower than that reported in previous studies.

Hypoxic-ischaemic encephalopathy of the newborn remains an important cause of mortality and morbidity in newborns^1^. The results of large randomised controlled trials (RCTs) indicate that therapeutic hypothermia (TH), using either selective head cooling (SHC) or whole body cooling (WBC), reduces the incidence of death and severe disability following neonatal encephalopathy^2^,^3^,^4^,^5^,^6^,^7^,^8^. Based on these studies, TH initiated within 6 h of birth has been recommended since 2010 as the standard of care for newborn infants ≥36 weeks gestation and ≥1,800 g birth weight with moderate to severe neonatal encephalopathy^9^,^10^. Now that the technique has become a routine part of clinical practice, it is expected that TH will be used in a broader range of neonates than the recommendations suggest^11^. Indeed, a survey conducted in 330 North American neonatal intensive care units demonstrated that approximately 30% of the units administered TH to neonates ≤35 weeks gestation^12^. An extensive registry established in the United Kingdom for the TOBY Study suggested the presence of “therapeutic drift”, whereby TH was being used outside the originally intended indications. In approximately 10% of neonates registered between December 2006 and July 2011, cooling commenced >6 h after birth, and the extent of birth asphyxia suggested by initial blood base deficits and amplitude-integrated encephalography (aEEG) patterns gradually became less severe over the study period^4^.

Previously in Japan, the use of TH as a clinical strategy outside clinical trials had been determined by empirical rather than evidence-based indications and protocols^13^. To ensure evidence-based best practice, a series of substantive alterations were undertaken in 2010^14^ to coincide with the release of the revised Consensus Statement and Treatment Recommendation on Cardiopulmonary Resuscitation (CoSTR)^9^. Owing to the nationwide campaign, adherence to evidence-based cooling protocols improved from 20.7% to 94.7% in less than 3 years^14^.

To further examine and monitor the use of TH in neonates, an online case registry was established in January 2012. The aims of this study were to examine adherence to the standard cooling protocol and illuminate changes in cooling practice during the first 3 years of the case registry.

## Results

### Clinical characteristics of registered patients

**Figure 1:**
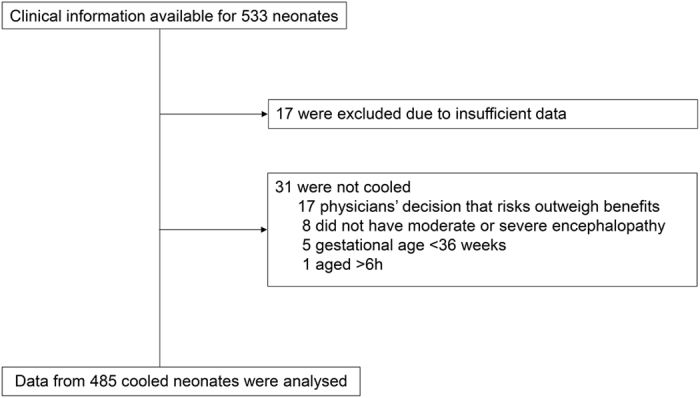
Profile of the registry.

Over the 3-year period, the clinical information of 533 infants was submitted from 100 units; 17 were excluded from the analysis because of missing data (Fig. 1). Thirty-one neonates of gestational age 38.7 ± 2.0 weeks and birth weight 2,889 ± 509 g were considered for TH, but were not cooled because: (i) physicians considered that the risk of TH outweighed the potential benefits because of the patient’s clinical condition (n = 17); (ii) encephalopathy was mild at the time of admission (n = 8); (iii) gestational age was <36 weeks (n = 5), or (iv) the neonate was aged >6 h (n = 1). The remaining 485 neonates of gestational age 38.7 ± 3.5 weeks and birth weight 2,862 ± 465 g underwent TH (Table 1). Data from non-cooled neonates were not subject to further analysis, and the proportions presented in the following section are calculated based on the 485 cooled neonates unless otherwise stated.

### Indications for cooling

Seventeen neonates (3.5%) were <36 weeks gestation, and three (0.6%) weighed <1,800 g. Apgar scores at 10 minutes were recorded for 369 neonates; scores ≤5 were present in 220 cases (59.6%). Severe acidosis (pH < 7 or base deficit ≥16 mmol/L) for the cord or first blood gas analysis within 1 h of birth was confirmed in 334 neonates (68.9%). The majority (469 neonates, 96.7%) had required persistent resuscitation for >10 minutes after birth. Four neonates (0.8%) neither required continuous resuscitation for >10 minutes, nor had severe acidosis or 10-minute Apgar scores ≤5; however, two had clinical seizure activity. Of 471 neonates with completed Sarnat encephalopathy stage data at admission, 288 (61.1%) and 128 (27.2%) neonates had stage II and III neonatal encephalopathy, respectively. Fifty-five (11.7%) had stage I encephalopathy; however, abnormal primitive reflexes (for example weak/absent sucking, rooting and the Moro reflex) were observed in all 55 cases, and signs suggestive of moderate encephalopathy, such as moderate/severe hypotonia (n = 21), clinical seizure activity (n = 4) and moderately/severely abnormal aEEG (n = 4) were observed in the majority. An aEEG was obtained in 295 (60.8%) and 201 (41.4%) neonates at admission and on the day of rewarming, respectively. The use of aEEG was more common for neonates who were cooled by WBC than those cooled by SHC at admission (66.1% for WBC *versus* 53.9% for SHC*, P* = 0.016) and on the day of rewarming (49.8% for WBC *versus* 30.0% for SHC, *P* < 0.001).

### Therapeutic hypothermia

**Figure 2:**
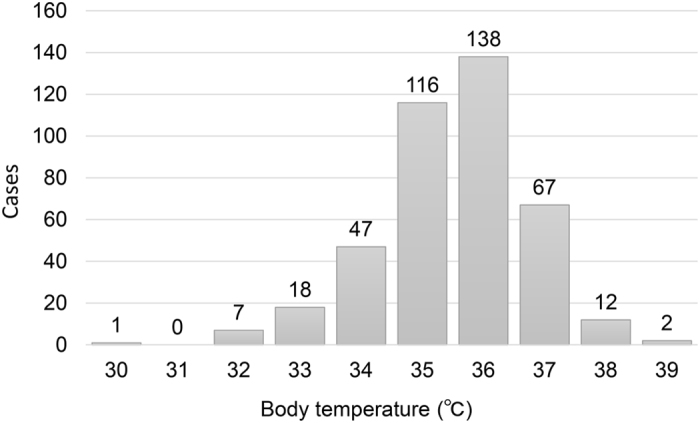
Mean body temperature at admission. The mean body temperature at admission was 35.9 °C ±1.5 °C (mean ± standard deviation. Body temperature was ≥38 °C, <35 °C and <33 °C in 14 (3.4%), 73 (17.9%) and eight neonates (2.0%), respectively. Data are based on the 408 neonates whose body temperature was recorded at admission.

In 408 neonates with temperature data available at admission, mean body temperature was 35.9 ± 1.5 °C (Table 2). Body temperature at admission was ≥38 °C in 14 (3.4%) neonates, whereas hypothermia (<35 °C) was recorded in 73 cases (17.9%, Fig. 2). In 468 cases with data available, cooling was commenced on average 222 ± 93 minutes after birth and 105 ± 87 minutes after admission. Cooling was commenced within 6 h of birth in 452 cases (96.6%). After the commencement of cooling, the target core body temperature was achieved in an average of 94 ± 154 minutes, leading to the completion of cooling initiation on average 312 ± 183 minutes after birth.

Among 476 neonates with complete data recorded over the first 4 days, SHC and WBC were used in 181 (38.0%) and 295 (62.0%) neonates, respectively. The mean core body temperature during cooling (from 6–72 h after the commencement of cooling) was 34.0 ± 0.6 °C and 33.7 ± 0.5 °C for SHC and WBC, respectively. The mean heart rates during SHC and WBC were 114 ± 15 beats/min and 112 ± 14 beats/min, respectively, and the mean blood pressure was 49 ± 6 mmHg and 48 ± 6 mmHg, respectively (Supplemental Table 1).

Adjuvant neuroprotective therapies were used in 148 cases (30.5%) using magnesium sulphate (n = 114), erythropoietin (n = 16), edaravone (n = 12), osmotic diuretics (n = 8), phenobarbital (n = 5) and/or ulinastatin (n = 3).

### Outcome at discharge

Of 474 neonates whose short-term outcome was confirmed, 430 (90.7%) were discharged home after 59.3 ± 122.3 days, 13 (2.7%) died before discharge and 31 (6.5%) were referred to other hospitals or transitional centres for further medical care (Table 3). The majority (86.3%) were successfully weaned from mechanical ventilation after 11.2 ± 27.0 days, whereas 46 (9.7%) required persistent mechanical ventilation, even at the time of discharge or transfer to another institution. Full oral feeding was established in 386 (81.4%) cases during initial hospital admission.

### Adverse events

Serious adverse events were observed in 371 cases, and consisted of: hypotension (34.8%); clinically diagnosed seizures (24.7%); coagulation disorders (13.2%); arrhythmia (1.4%); hypoglycaemia (1.0%); septicaemia (0.8%) and subcutaneous fat necrosis (0.4%) (Table 3).

### Changes in practice over 3-year study period

Apgar score at 10 minutes, cord or first blood pH and base deficit, Sarnat encephalopathy stage and Thompson encephalopathy score at admission did not change significantly in the first 3 years of the registry (Table 1). Similarly, body temperature at admission and the time to start cooling (after birth) remained unchanged over the 3 years (Table 2). In contrast, the mean time to reach the target temperature after initiation of cooling fell significantly from 104 ± 141 minutes in 2012 to 66 ± 71 minutes in 2014 (*P* = 0.004). The proportion undergoing SHC fell and the proportion undergoing WBC rose over time, so that WBC accounted for 81.6% of cases in 2014 (*P* < 0.001 compared with 2012). In 2012, the proportion of neonates undergoing aEEG at admission was 44.1%, with 27.4% undergoing aEEG on the day of rewarming, which increased to 79.3% and 46.3%, respectively, in 2014 (both *P* < 0.001).

Short-term outcomes, including duration of mechanical ventilation, duration of hospital stay, requirement for tube feeding at discharge, requirement for mechanical ventilation at discharge or referral to other centres, and mortality rate during the initial hospital stay remained unchanged over the study period (Table 3).

## Discussion

Adherence to standard cooling protocols was maintained at a high level in Japan, even after the conclusion of the nationwide implementation campaign that ran between 2010 and 2012^14^. During the first 3 years of the Baby Cooling Register, there was a substantial change in cooling technique from SHC to WBC. A consistent trend towards more prompt initiation of cooling was also observed. The mortality rate of cooled neonates before discharge was 2.7%, substantially lower than previous clinical trials and registers^2^,^3^,^4^.

Despite the established efficacy of TH for neonatal encephalopathy^15^, approximately 40% of neonates do not respond, and consequently many will go on to develop permanent neurological impairments or succumb^16^. To further improve the cooling protocol, a recent large-scale RCT^17^ examined the potential benefits of deep (32.0 °C) and prolonged (120 h) hypothermia; however, there was a trend towards increased short-term mortality rates with deep/prolonged cooling compared with the current standard cooling protocol. Several other RCTs of TH with revised cooling indications/protocols are underway, but further improving the therapeutic efficacy of TH might be challenging considering the body of clinical evidence that has informed the choice of the current standard cooling criteria and protocol. Nevertheless, large-scale clinical databases of cooled neonates can now be established relatively easily, which may help identify novel techniques or refine cooling strategies that further improve outcomes in cooled neonates. The UK TOBY Register and the Vermont Oxford Network Neonatal Encephalopathy Registry were the first major attempts to collect a large-scale clinical dataset^18^,^19^. Although these registries recorded that the standard TH protocol was generally followed outside RCTs, the application of TH to neonates with less severe asphyxia, to those <36 weeks gestational age and/or >6 h after birth, was already evident. A more recent survey in California found that the proportion of neonates with mild encephalopathy who were cooled increased from 38% to 55% between 2010 and 2012^20^.

In contrast with the therapeutic drift observed in Western countries^18^,^19^,^20^, we found that, even up to 4 years after the initial recommendation of TH for neonatal encephalopathy^9^, the standard cooling criteria and protocol were followed in most neonatal care centres in Japan. When TH was newly recommended as a standard of care in 2010^9^, a substantial proportion of Japanese neonatal intensive care centres had already started cooling encephalopathic neonates according to empirically-acquired cooling indications and protocols^13^. To disseminate evidence-based best practice for TH for newborn infants, a dynamic nationwide campaign was conducted between 2010 and 2012, leading to a dramatic improvement in adherence to the standard cooling criteria and protocol^14^. The Baby Cooling Registry of Japan was opened in 2012 to disseminate evidence-based cooling practice by monitoring the clinical use of TH, and by giving feedback to participating units. As well as ensuring adherence to the standard cooling protocol, we found that cooling was initiated more promptly over the first 3 years of the registry, which may explain the increase that we observed in the number of neonates in whom TH was achieved within the therapeutic time window.

Our study also identified an increase in the use of WBC over time. Although SHC is non-inferior to WBC in its therapeutic benefit, it is more difficult to perform cot-side examinations, such as cranial ultrasound and electroencephalogram, when SHC is used. This might explain our observation that a smaller proportion of SHC-cooled neonates underwent aEEG compared with those cooled by WBC. Moreover, the concept of simultaneous head cooling and body warming is sometimes misleading. Indeed, our previous nationwide survey in Japan found that a substantial proportion of units were undertaking SHC using relatively warm cap temperatures >25 °C, which were servo-controlled with reference to the nasopharyngeal temperature^13^. As this empirical protocol was in widespread use in Japan by 2010, the Neonatal Hypothermia Task Force Japan decided to promote WBC rather than disseminating the correct protocol for SHC. This is likely to have contributed to the reduction in the use of SHC over time.

With regard to the short-term outcome of cooled neonates, it is known that the mortality rates of cooled neonates vary between RCTs, as seen in the CoolCap (33%)^2^, NICHD (24%)^3^ and UK TOBY (26%) studies^4^, despite the use of similar inclusion criteria. A more recent clinical study showed a relatively lower short-term mortality rate of 7% in neonates who were randomised to WBC to 33.5 °C for 72 h^17^. In our cohort, the mortality rate of cooled neonates was 2.7%. This might not fully be explained by the difference in severity of neonatal encephalopathy considering that standard cooling indications were followed closely. Global comparative studies are needed to illuminate the factors associated with short- and long-term outcomes of cooled neonates.

Several limitations of the current study must be acknowledged. First, we were not able to analyse follow-up data of our cohort of cooled neonates. For most participating units, only one or two cases were cooled each year, which will likely influence the level of experience within each unit, and the subsequent outcome of cooled neonates. The Baby Cooling Registry of Japan is currently collecting data after hospital discharge to investigate the outcome of neonates cooled in different types of units using various therapeutic regimens. Second, although we provided reference values for the heart rate and blood pressure in neonates cooled with SHC and WBC, we did not collect information regarding the use of inotropes. Third, the number of registered cases is smaller than might be expected from the approximately 1,000,000 births in Japan *per annum* that were registered between 2012 and 2014. We would expect to see 1,000–2,000 cases of neonatal encephalopathy each year given that the incidence of neonatal encephalopathy is approximately 1–2 per 1,000 live births in developed countries^21^, but just over 500 cases were recorded in the registry over 3 years. Hayakawa and colleagues have, however, estimated that the incidence of moderate to severe neonatal encephalopathy in Japan is 0.37 per 1,000 live births^22^. Considering that 219 of 287 registered level II-III neonatal intensive care centres participated in the current registry (76.3%), our database is likely to have included the majority of eligible neonates. Nevertheless, caution is required to avoid drawing conclusions from studies based on case registries, which may be influenced by selection bias.

In conclusion, a national registry of TH in neonatal encephalopathy has been successfully established in Japan and maintained for 3 years. During this period, TH was applied and conducted in close concordance with the criteria and protocols used in previous large-scale RCTs. There was some improvement in practice, such as the time required for the initiation of TH. The mortality rate during the initial hospital stay was considerably lower than previous reports. International comparative studies may help identify factors associated with short-term outcome that would help investigators refine and improve the TH protocol.

## Methods

This study was conducted in compliance with the Declaration of Helsinki. The protocols of the registry were approved by the Ethics Committees of Kurume University School of Medicine and Saitama Medical University, Japan. Since no patient identifiers were or are collected, the Ethics Committees advised that there is no statutory requirement for parental consent for data collection, and consent was not sought for the current registry.

The Neonatal Hypothermia Task Force Japan was formed by the Japan Society of Perinatal and Neonatal Medicine (JSPNM) and the Clinical Guidelines Committee for Neonatal Resuscitation in Japan (Neonatal Research Network Japan, Ministry of Health, Labour and Welfare [MHLW]) in June 2010 to implement evidence-based TH practice in Japanese neonatal intensive care centres. The Task Force launched an online case registry to monitor newborn TH practice in January 2012. The TH strategy was derived from the JSPNM and MHLW Japan Working Group Practice Guidelines Consensus Statement, which in turn had been developed by summarising and integrating the indications and cooling protocols used in large-scale RCTs^2^,^3^,^4^. All Japanese level II or III neonatal intensive care centres registered as designated hospitals for postgraduate clinical training with the Japanese Society for Neonatal Health and Development were invited to join the registry. Basic, mandatory clinical information for each case was input online via the official website of the Baby Cooling Registry of Japan. A unique identification number was allocated to each registered case. Data were anonymised so that patients could not be identified, obviating the need for individual consent for data collection. Although the registry guidance emphasised the importance of observing standard cooling protocols, participating units were requested to register all neonates (i) who underwent TH regardless of adherence to the guidance, and (ii) who were referred to the unit for consideration of TH but ultimately did not undergo cooling.

Case record forms were developed based on the format used in the UK TOBY Register^4^, including patient characteristics, clinical condition at birth, severity of encephalopathy assessed by established scales^23^,^24^, patterns of aEEG^25^, core body temperature, cardiovascular and respiratory parameters, supportive treatments, use of sedatives and analgesics, clinical complications, and short-term outcomes at hospital discharge. Adverse events were reported in accordance with the guidance and protocol provided on the official website of the Baby Cooling Registry of Japan (Supplemental Table 2). Participating units were encouraged to obtain magnetic resonance images before discharge and perform developmental assessments at around 12 months and 2 years of age.

### Statistical analysis

In this observational study, descriptive data analysis was performed for the dataset compiled during the first 3 years of the registry (between 1^st^ January 2012 and 31^st^ December 2014). For the current analysis, only data collected during the primary hospital stay were analysed. Each record was inspected for case duplication, apparent input errors or excessive unexplained missing data (>5% of the individual dataset without plausible explanations). To assess changes in patient characteristics and practice with time, data were grouped into 12-month periods according to the date of birth of each neonate, and the data from 2013 and 2014 were compared with those from 2012 using either the chi-squared test or Student’s t-test with the Bonferroni correction. Values are shown as number (proportion, %) for categorical variables or mean ± standard deviation for normally distributed continuous variables.

## Additional Information

**How to cite this article**: Tsuda, K. *et al*. Therapeutic hypothermia for neonatal encephalopathy: a report from the first 3 years of the Baby Cooling Registry of Japan. *Sci. Rep.* **7**, 39508; doi: 10.1038/srep39508 (2017).

**Publisher's note:** Springer Nature remains neutral with regard to jurisdictional claims in published maps and institutional affiliations.

## Supplementary Material

Supplementary Information

## Figures and Tables

**Table 1:** Maternal and neonatal baseline characteristics by year of registration.

Variables	2012	2013	2014	Entire period
Number of registrations	186	154	145	485
Centres registering cases	73	71	59	100
Maternal				
Age (year)	31.7 ± 5.1	32.4 ± 5.2	31.5 ± 5.6	31.8 ± 5.3
Primigravida	67 (36.2)	60 (39.7)	50 (35.0)	177 (37.0)
Emergency delivery*	136 ± 73.1	105 ± 68.2	109 ± 75.2	350 ± 72.2
Neonatal				
Gestational age (week)	39.0 ± 1.7	38.7 ± 3.5	38.3 ± 4.9	38.7 ± 3.5
Birth weight (g)	2839 ± 460	2904 ± 443	2847 ± 495	2862 ± 465
Outborn	132 (71.0)	110 (71.4)	100 (69.0)	342 (70.5)
Apgar score				
1 min.	1 {1–3}	2 {1–3}	1 {1–2}	1 {1–3}
5 min.	4 {2–5}	3 {2–5}	3 {2–5}	4 {2–5}
10 min.	5 {3–6}	5 {3.5–7}	5 {3–7}	5 {3–7}
Cord or first blood gas <1 h of birth				
pH	6.95 ± 0.21	6.95 ± 0.22	6.92 ± 0.20	6.94 ± 0.21
Base deficit (mmol/L)	14.8 ± 11.1	14.0 ± 0.18	15.1 ± 10.1	14.6 ± 10.5
Sarnat encephalopathy stage at admission				
Stage I	20 (11.2)	12 (8.0)	23 (16.2)	55 (11.7)
Stage II	110 (61.5)	97 (64.7)	81 (57.0)	288 (61.1)
Stage III	49 (27.4)	41 (27.3)	38 (26.8)	128 (27.2)
Thompson encephalopathy score at admission				
	11 {8–15}	11 {9–15}	11 {7–15}	11 {8–15}

Data are processed excluding missing values and are presented as number (%), mean ± standard deviation or median {inter-quartile range}.

^*^Including emergency caesarean, forceps and vacuum-assisted vaginal delivery.

**Table 2:** Initiation of cooling by year of registration.

Variables	2012	2013	2014	Entire period
Selective head cooling	101 (54.6)	54 (36.0)	26 (18.4)	181 (38.0)
Whole body cooling	84 (45.4)	96 (64.0)	115 (81.6)*	295 (62.0)
Body temperature at admission (°C)	36.0 ± 1.1	35.9 ± 1.9	35.8 ± 1.4	35.9 ± 1.5
Time of admission after birth (min.)	102 ± 73	114 ± 88	107 ± 90	107 ± 83
Commencement of cooling after admission (min.)	101 ± 80	104 ± 94	113 ± 88	105 ± 87
Commencement of cooling after birth (min.)	215 ± 92	226 ± 93	225 ± 93	222 ± 93
Time to reach the target temperature after the commencement of cooling (min.)	104 ± 141	110 ± 216	66 ± 71**	94 ± 154
Time to reach the target temperature after birth (min.)	316 ± 179	331 ± 234	288 ± 125	312 ± 183

Values are shown as number (%) or mean ± standard deviation.

*P < 0.001 from the chi-squared test with the Bonferroni correction, compared with 2012.

**P = 0.004 from the analysis of variance and Dunnett’s test, compared with 2012.

Target core-body temperatures were 34.5 ± 0.5 °C for selective head cooling and 33.5 ± 0.5 °C for whole body cooling.

**Table 3:** Short-term outcomes and adverse events by year of registration.

Variables	2012	2013	2014	Entire period
Adverse events during hospital stay	n = 186	n = 154	n = 145	n = 485
Hypotension†	64 (34.4)	48 (31.2)	57 (39.3)	169 (34.8)
Clinically diagnosed seizures	39 (21.0)	38 (24.7)	43 (29.7)	120 (24.7)
Coagulation disorders††	17 (9.1)	25 (16.2)	22 (15.2)	64 (13.2)
Arrhythmia‡	2 (1.1)	3 (1.9)	2 (1.4)	7 (1.4)
Hypoglycaemia‡‡	1 (0.5)	2 (1.3)	2 (1.4)	5 (1.0)
Culture-positive septicaemia	0 (0.0)	2 (1.3)	2 (1.4)	4 (0.8)
Subcutaneous fat necrosis	1 (0.5)	1 (0.6)	0 (0.0)	2 (0.4)
Short-term outcome during hospital stay*	n = 184	n = 150	n = 140	n = 474
Successful weaning from mechanical ventilation	156 (84.8)	135 (90.0)	118 (84.3)	409 (86.3)
Duration of mechanical ventilation (day)**	11.2 ± 31.8	9.2 ± 13.0	13.3 ± 31.2	11.2 ± 27.0
Establishment of full oral feeding (day)**	15.9 ± 16.8	14.1 ± 8.9	13.1 ± 8.0	14.5 ± 12.4
Duration of hospital stay (day)*	72.7 ± 167.4	47.1 ± 69.6	54.5 ± 90.0	59.3 ± 122.3
Status of discharge*	n = 184	n = 150	n = 140	n = 474
Death before discharge	5 (2.7)	3 (2.0)	5 (3.6)	13 (2.7)
Discharged home	168 (91.3)	137 (91.3)	125 (89.3)	430 (90.7)
Transferred to a different hospital	11 (6.0)	10 (6.7)	10 (7.1)	31 (6.5)
Dependence on tube feeding	38 (20.7)	22 (14.7)	28 (20.0)	88 (18.6)
Dependence on respiratory support	19 (10.3)	12 (8.0)	15 (10.7)	46 (9.7)

Values are shown as the number (%) or mean ± standard deviation.

^*^Excluding neonates whose clinical outcome has not been confirmed.

^**^Excluding neonates who were mechanically ventilated or fed through a tube at discharge.

^†^Defined as persistent hypotension with mean blood pressure ≤40 mmHg.

^††^Defined as clinical bleeding, thrombocytopaenia and/or abnormal coagulation studies.

^‡^Defined as persistent or recurrent arrhythmia, excluding sinus bradycardia.

^‡‡^Defined as blood glucose <45 mg/dL.
