# Supplementary material for: Therapeutic hypothermia for neonatal encephalopathy: a report from the first 3 years of the Baby Cooling Registry of Japan
Source: Sci Rep. 2017 Jan 4;7:39508. doi: 10.1038/srep39508 (PMC5209702; doi:10.1038/srep39508)
Supplement: Supplementary Information [file srep39508-s1.doc]

**Title:**

**Therapeutic hypothermia for neonatal encephalopathy: a report from the first 3 years of the Baby Cooling Registry of Japan**

**Short title:** Neonatal hypothermia in Japan

**Authors:**

Kennosuke Tsuda^1^, Takeo Mukai^2^, Sachiko Iwata^1,3^, Jun Shibasaki^4^, Takuya Tokuhisa^5^, Tomoaki Ioroi^6^, Hiroyuki Sano^7^, Nanae Yutaka^7^, Akihito Takahashi^8^, Akihito Takeuchi^9^, Toshiki Takenouchi^10^, Yuko Araki^11^, Hisanori Sobajima^12^, Masanori Tamura^12^, Shigeharu Hosono^13^, Makoto Nabetani^7^, and Osuke Iwata^1,3^ on behalf of The Baby Cooling Registry of Japan Collaboration Team.

**Affiliations:**

1. Department of Paediatrics and Child Health, Kurume University School of Medicine, Fukuoka, Japan

2. Division of Molecular of Therapy, Center for Advanced Medical Research, The Institute of Medical Science, The University of Tokyo, Tokyo, Japan

3. Centre for Developmental and Cognitive Neuroscience, Kurume University School of Medicine, Fukuoka, Japan

4. Department of Neonatology, Kanagawa Children's Medical Center, Kanagawa, Japan

5. Division of Neonatology, Perinatal Medical Center, Kagoshima City Hospital, Kagoshima, Japan

6. Department of Pediatrics, Perinatal Medical Center, Himeji Red Cross Hospital, Hyogo, Japan

7. Department of Pediatrics, Yodogawa Christian Hospital, Osaka, Japan

8. Department of Pediatrics, Kurashiki Central Hospital, Okayama, Japan

9. Division of Neonatology, National Hospital Organization Okayama Medical Center, Okayama, Japan

10. Department of Pediatrics, Keio University School of Medicine, Tokyo, Japan

11. Faculty of Informatics, Shizuoka University, Hamamatsu, Shizuoka, Japan

12. Division of Neonatology, Center for Maternal, Fetal and Neonatal Medicine,

Saitama Medical Center, Saitama Medical University, Saitama, Japan

13. Division of Neonatology, Nihon University Itabashi Hospital, Tokyo, Japan

**Correspondence to:**

Dr Osuke Iwata

Centre for Developmental and Cognitive Neuroscience, Kurume University School of Medicine

67 Asahimachi, Kurume, Fukuoka, 830-0011 Japan.

E-mail: o.iwata@ucl.ac.uk Tel: +81 942 31-7565 Fax: +81 942 38-1792

**Online Supplemental Table 1:**

**Heart rate and blood pressure during therapeutic hypothermia**

**A: Selective head cooling**

| **n=181** | | **Hours from the commencement of cooling** | | | | |
| --- | --- | --- | --- | --- | --- | --- |
|  | Percentile | 0-<6h | 6-<24h | 24-<48h | 48-<72h | 72-<96h |
| **Heart rate**  **(bpm)** | 1^st^ | 87 | 81 | 76 | 70 | 77 |
|  | 5^th^ | 94 | 89 | 89 | 83 | 87 |
|  | 10^th^ | 100 | 92 | 93 | 90 | 93 |
|  | 50^th^ | 127 | 119 | 113 | 108 | 115 |
|  | 90^th^ | 157 | 144 | 137 | 131 | 140 |
|  | 95^th^ | 165 | 150 | 144 | 138 | 148 |
|  | 99^th^ | 190 | 158 | 156 | 144 | 159 |
| **Mean blood pressure (mmHg)** | 1^st^ | 25 | 28 | 32 | 35 | 36 |
|  | 5^th^ | 30 | 33 | 37 | 40 | 40 |
|  | 10^th^ | 34 | 37 | 39 | 43 | 43 |
|  | 50^th^ | 47 | 46 | 48 | 51 | 50 |
|  | 90^th^ | 60 | 58 | 59 | 64 | 62 |
|  | 95^th^ | 62 | 63 | 63 | 68 | 69 |
|  | 99^th^ | 70 | 71 | 71 | 77 | 85 |

**B: Whole body cooling**

| **n=295** | | **Hours from the commencement of cooling** | | | | |
| --- | --- | --- | --- | --- | --- | --- |
|  | Percentile | 0-<6h | 6-<24h | 24-<48h | 48-<72h | 72-<96h |
| **Heart rate**  **(bpm)** | 1^st^ | 80 | 77 | 78 | 79 | 82 |
|  | 5^th^ | 90 | 85 | 85 | 85 | 94 |
|  | 10^th^ | 94 | 90 | 90 | 89 | 99 |
|  | 50^th^ | 121 | 113 | 111 | 109 | 119 |
|  | 90^th^ | 149 | 140 | 135 | 131 | 148 |
|  | 95^th^ | 157 | 145 | 142 | 138 | 155 |
|  | 99^th^ | 177 | 156 | 151 | 149 | 170 |
| **Mean blood pressure (mmHg)** | 1^st^ | 24 | 28 | 30 | 34 | 34 |
|  | 5^th^ | 32 | 34 | 36 | 38 | 38 |
|  | 10^th^ | 35 | 38 | 39 | 41 | 40 |
|  | 50^th^ | 46 | 47 | 47 | 49 | 49 |
|  | 90^th^ | 57 | 58 | 58 | 59 | 61 |
|  | 95^th^ | 61 | 62 | 61 | 63 | 65 |
|  | 99^th^ | 69 | 70 | 66 | 70 | 73 |

Abbreviations: bpm, beat per minute.

**Online Supplemental Table 2: Information collected for the Baby Cooling Registry of Japan**

Section 1 (all cases):

| Transport data |  |
| --- | --- |
| Date of birth (years and month) | □□/□□ |
| Birth hospital | - Outborn □ Inborn |
| Transport method | □ Ground transport □ Air transport  □ Sea transport □ Other |
| Transport team | □ Paediatrician □ Obstetrician  □ Nurse □ Ambulance crew only |
| Body temperature before departure | □□.□ °C |
| Age at admission to the referral centre | □□:□□ |
| Body temperature at admission | □□.□ °C |

| Obstetric-perinatal history |  |
| --- | --- |
| Maternal age at delivery | □□ years old |
| Parity | - Primigravida □ Multigravida |
| Pregnancy complications | □ Preeclampsia or eclampsia  □ Gestational diabetes mellitus  □ Epilepsy □ Thyroid malfunction  □ Placenta praevia  □ Medication during pregnancy |
| Mode of delivery | □ Vaginal birth, unassisted  □ Vaginal birth, assisted  □ Caesarean section, elective  □ Caesarean section, emergency |
| Delivery complications | □ Prolapsed cord  □ Placental abruption  □ Shoulder dystocia  □ Ruptured uterus  □ Head entrapment  □ Vaginal breech |

| Clinical details of baby at birth |  |
| --- | --- |
| Gestational age at birth | □□ weeks □ days |
| Birth weight | □□□□ g |
| Length | □□.□ cm |
| Head circumference | □□.□ cm |
| Number of offspring | □ Single □ Twins (Monozygotic)  □ Twins (Dizygotic) □ Triplets |
| Apgar scores | 1 min. □□ 5min. □□ 10min. □□ |
| First spontaneous breathing after birth | □□ min. |
| Continued resuscitation over 10 min. | - Yes □ No |
| Blood gas findings within 60 min. after birth | pH □.□□□  Base deficit □□.□ mmol/L  pO_2_ □□□ mmHg pCO_2_ □□□ mmHg  Lactate □□.□ mmol/L |
| Congenital anomalies | - Yes □ No |
| Systemic complications at admission | - Yes □ No |
| Modified Sarnat encephalopathy stage at admission | - Stage I □ Stage II □ Stage III |
| aEEG prior to cooling | - Yes □ No   Initial findings (Background):   - Normal   (upper margin>10, lower margin>5)   - Moderately abnormal   (upper margin>10, lower margin<5)   - Severely abnormal   (upper margin<10, lower margin<5)  □ Seizure pattern  □ N/A |
| Exclusion criteria | □ Aged >6h  □ Gestational age <36 weeks  □ Birth weight <1800g  □ Severe congenital malformation  □Physicians’ decision that risks outweigh benefits  □ Lack of resources |

Section 2 (cooled cases only):

| Cooling and supportive treatments |  |
| --- | --- |
| Commencement of cooling after birth | □□ h □□ min. |
| Cooling modality | □ Selective head cooling  □ Whole body cooling  □ Other |
| Cooling equipment used | □ Arctic Sun  □ Medi-Cool  □ Medi-Therm  □ Other |
| Primary site for body temperature monitoring | □ Rectum  □ Oesophagus  □ Nasopharynx  □ Other |
| Blood gas analysis | □ Measurement at 37°C (alpha-stat)  □ Measurement at the patient’s body temperature (pH-stat) |
| Sedatives and anticonvulsants | □ Phenobarbital  □ Midazolam  □ Phenytoin  □ Other |
| Combination therapy with hypothermia | - Yes □ No |
| Significant adverse events during therapeutic hypothermia | □ Hypotension  □ Clinically diagnosed seizures  □ Coagulation disorders  □ Arrhythmia  □ Hypoglycaemia  □ Septicaemia  □ Subcutaneous fat necrosis  □ Other |

| Discharge status |  |
| --- | --- |
| Outcome | □ Discharged home  □ Transferred to another hospital  □ Died  Post-mortem examination   - Yes □ No |
| Length of Hospital stay | □□ days |
| Length of mechanical ventilation | □□ days |
| Establishment of full oral feeding | □□ days |
| Special feeding support at discharge | □ Yes □ No  □ Enteral nutrition  □ Gastrostomy nutrition  □ Other |
| Tracheostomy at discharge | - Yes □ No   Discharged with a ventilator   - Yes □ No |
| Medication at discharge | - Yes □ No |
| Age at MRI scan | - Yes □ No   　□□ days |

Questionnaires (originally distributed in Japanese) are presented after translation into English for convenience.

Serious adverse events were defined as follow. Hypotension: persistent hypotension with mean blood pressure ≤40mmHg. Seizures: clinically diagnosed seizures with/without electrographic correlates. Coagulation disorders: clinical bleeding, thrombocytopaenia and/or abnormal clotting studies. Arrhythmia: persistent or recurrent arrhythmia excluding sinus bradycardia. Hypoglycaemia: blood glucose <45mg/dL. Septicaemia: blood-culture-positive septicaemia. Subcutaneous fat necrosis: firm, erythematous nodules and plaques over the trunk, arms, buttocks, thighs and cheeks.
